# Invariant categorical color regions across illuminant change coincide with focal colors

**DOI:** 10.1167/jov.23.2.7

**Published:** 2023-02-13

**Authors:** Takuma Morimoto, Yasuki Yamauchi, Keiji Uchikawa

**Affiliations:** 1 Department of Psychology, Justus-Liebig-Universität Giessen, Giessen, Germany; 2 Department of Experimental Psychology, University of Oxford, Oxford, UK; 3 Department of Informatics and Electronics, Yamagata University, Yamagata, Japan; 4 Human Media Research Center, Kanagawa Institute of Technology, Atsugi, Japan; 5 Department of Information Processing, Tokyo Institute of Technology, Yokohama, Japan

**Keywords:** categorical color perception, categorical color constancy, focal color, asymmetric matching, OSA perceptually uniform color scale

## Abstract

Are there regions in a color space where color categories are invariant across illuminant changes? If so, what characteristics make them more stable than other regions? To address these questions, we asked observers to give a color name to 424 colored surfaces, presented one at a time, under various chromatic illuminants. Results showed a high degree of categorical color constancy, especially under illuminants that occur in the natural environment. It was also shown that surfaces selected as a focal color (the best example of a color category) are more resistant to illuminant change than nonfocal color samples. We additionally ran an asymmetric color matching experiment to quantify the shift of color appearance induced by illuminant changes using surfaces that were all named gray, thereby disentangling the appearance-based color constancy from the categorical color constancy (which are often confounded). Results suggested that the appearance of color samples largely shifted owing to illuminant changes, even though all samples were named gray; showing that the constancy of a color category is substantially more robust than the constancy of color appearance.

## Introduction

Physical parameters underlying color variation are continuous, and the associated subjective experience also varies continuously from one color to another. Yet, at the same time color perception can be discrete: we can count how many colors there are in a rainbow. This discrete nature is known as categorical color perception, and it offers a prime example to use colors in a daily life. Importantly, our visual system flexibly uses such a continuous–discrete duality to achieve diverse visual tasks from discriminating subtle color differences to effectively communicating with others using color (e.g., can you pass me the red coat?).

The interdisciplinary nature of color categories has naturally attracted a wide range of fields including biology, psychology, and linguistics. In the long history of research, many critical questions have been raised, investigated, and discussed. Are there basic color categories in individual cultures (Berlin & Kay, 1969)? How are they different or shared across cultures (Uchikawa & Boynton, 1987), and are there universal constraints on the formation of color categories (World Color Survey; Kay & Cook, 2016; Kay & Regier, 2003, 2007; Lindsey & Brown, 2006, 2009)? Do the linguistic labels we give to colored stimuli affect their color appearance (the Sapir–Whorf hypothesis; Kay & Kempton, 1984)? Is it necessary to have linguistic labels to perform color categorization (Siuda-Krzywicka et al., 2019)? Does the strength of categorical effects differ between the right and the left visual field (Drivonikou et al., 2007; Gilbert, Regier, Kay, & Ivry, 2006; Gilbert, Regier, Kay, & Ivry, 2008; but also see Brown, Lindsey, & Guckes, 2011; Suegami, Aminihajibashi, & Laeng, 2014; Webster & Kay, 2012; Witzel & Gegenfurtner, 2011; Witzel & Gegenfurtner, 2016)? Moreover, many psychophysical studies have demonstrated functional benefits to having color categories (Witzel & Gegenfurtner, 2018; Witzel, 2019). One representative example is the reduction of response time in a suprathreshold discrimination task when discriminating between colors in different color categories compared with the discrimination of colors within the same category (Bornstein & Korda, 1984). A cross-language study went further and showed that this categorical facilitation effect can be specific to the language that individuals use (Winawer et al., 2007). Curiously, certain color categories emerge only in certain conditions. The terms gold or silver are used only for a glossy surface that exhibits specular reflection (Okazawa, Koida, & Komatsu, 2011) or the term brown is used only to describe the color of a surface, not the color of a light (Buck et al., 2016; Uchikawa, Uchikawa, & Boynton, 1989). In addition to these studies on the perceptual basis of categorical color perception, the demand of communication has been suggested to affect the geometry of color categories, showing the cultural influences on the formation of color categories (Davidoff, 2001; Gibson et al., 2017; Mylonas et al., 2022; Steels & Belpaeme, 2005). In addition, color classification and categorization tasks are major interests in computer vision (Parraga & Akbarini, 2020). A recent study reported that an artificial visual system forms human-like color categorical boundaries as a result of learning to recognize an object in images (de Vries, Akbarinia, Flachot, & Gegenfurtner, 2022).

These studies brought the whole picture of categorical color perception into sharp focus from wide perspectives. However, a crucial but missing element in these past studies is that, in the real world, lighting environments largely change over space and time. For color categories to be useful in everyday tasks, one category should not easily change to another when a scene illuminant changes. Some studies have explicitly measured category-based color constancy. Uchikawa, Uchikawa, and Boynton (1989) might be the first study that introduced a color naming task to study color constancy, where the effect of chromatic adaptation was investigated. Troost and Weert (1991) replicated a Mondrian experiment conducted by Arend and Reeves (1986) using both color naming and color matching tasks. They emphasized that color naming is potentially more suitable than color matching as a measure of color constancy because it is more naturalistic and, therefore, easier for observers to perform. Furthermore, Speigle and Brainard (1996) showed that the effects of illuminant change on color appearance are highly consistent across achromatic adjustment, asymmetric matching, and color naming. Hansen, Walter, and Gegenfurtner (2007) investigated the independent contributions of the spatial and temporal contexts on categorical color constancy. More recently, a series of studies measured color naming patterns using color patches under simulated illuminant changes on a computer monitor (Olkkonen, Hansen & Gegenfurtner, 2009), Munsell color chips presented under real illuminations (Olkkonen et al., 2010), and solid three-dimensional objects under real illuminations (Ling, Allen-Clarke, Vurro, Hurlbert, 2008). It was shown that categorical color constancy holds slightly better for real surfaces than simulated surfaces, but, more important, these studies agreed that humans perform robust color categorization under different illuminants. Moreover, categorical response patterns were consistent across observers. One experiment conducted using a highly naturalistic experimental setup also supported the robust categorical identification of colored papers under several lighting environments (Granzier, Brenner, & Smeets, 2009). Most recently, Ma, Liao, Yan, and Shinomori (2018) used a three-channel LED and showed that categorical constancy holds generally better for red and green illuminants than for blue and yellow illuminants.

Basic aspects of categorical color constancy were uncovered by these efforts. Yet, considering the large volumes of studies on color constancy (Foster, 2011; Hurlbert, 2019; Smithson, 2005), our knowledge on its categorical aspect is still largely limited.

We, therefore, conducted two types of psychophysical experiments to further investigate the mechanisms underpinning categorical color constancy, using real surfaces and real illuminants. Preliminary results and analysis of this study were presented at past research conferences (Uchikawa, Emori, Toyooka, & Yokoi, 2002; Uchikawa, Yokoi, & Yamauchi, 2004), and this article provides the full contents of the study. Aside from the potentially better constancy for real surfaces than for two-dimensional stimuli, the use of real stimuli has an advantage in that the stimulus range is not constrained by the color gamut of a monitor, especially when stimuli are presented under highly saturated illuminants. We leveraged this strength and measured color naming patterns under a wider range of illuminants than past studies, including illuminants close to the spectrum locus. The first experiment was to name each of 424 color samples, presented one at the time on a gray surround placed under a colored test illuminant, using 1 of 11 basic color terms. We were particularly interested in i) whether any regions in a color space remain invariant in their color category against illuminant changes and if so ii) what makes those regions more stable than others. Such invariant colors could serve particularly important roles in visual tasks that might heavily rely on color categories. In a second experiment, we measured how much the color appearance of the surfaces named by the same color category shifts in response to illuminant color changes. In a hypothetical scenario where color appearance does not change at all thanks to the appearance-based color constancy, naturally, color categories should not change either. Past studies have extensively investigated the nature of this distinction between the color appearance of an object and the intrinsic surface color associated with underlying physical property of the object that stays constant regardless of the change of surrounding lighting environments (Arend & Reeves, 1986; Radonjic & Brainard, 2016; Reeves, Amano, & Foster, 2008). It was repeatedly shown that observers can differentiate such distinctions and that color constancy works better when observers are explicitly instructed to judge the latter surface color. Categorical color perception is a naturalistic task, and we hypothesized that when observers were simply asked to judge a color category of a given surface, they based their judgments on surface color rather than color appearance even though they were not explicitly instructed to do so. Our second experiment was performed to disentangle two types of color constancy mechanisms which are often confounded in previous studies.

## General methods

### Test surface

**Figure 1.**
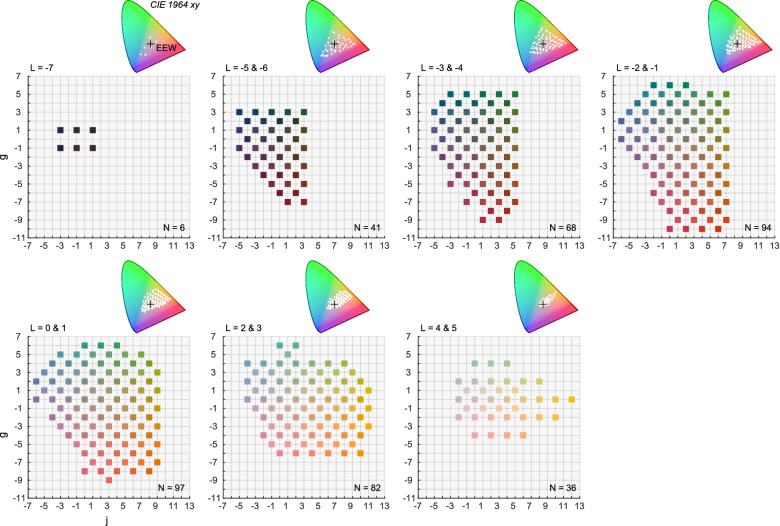
OSA uniform color system (OSA-UCS) used in all experiments. Each panel shows chromatic coordinates of color samples at different lightness levels. Inserted xy chromaticities were calculated under equal energy white illuminant.

All experiments used 424 color samples developed by the Optical Society of America (OSA, currently Optica), known as OSA uniform color scales (OSA-UCS). Each color sample has three coordinates *j*, *g*, and *L* which stand for *jaune* (meaning yellow), green, and lightness, respectively. A perceptual color difference between a color pair in OSA-UCS is defined as an Euclidean distance in *j*, *g*, and √2*L* three-dimensional space (i.e., the *L* axis should be weighted by the factor of √2). Figure 1 shows the coordinates of all color samples. Each subpanel combines samples at two lightness levels (except for the plane of *L* = −7). At even lightness planes, the chromatic coordinates are all even integers by the design of the color system. In the same way at odd lightness planes, chromatic coordinates are always odd integers. For this reason there are no overlapping data points in Figure 1. We measured the spectral reflectance of each color sample using a spectrometer from 400 nm to 720 nm in 10 nm steps (X-Rite, Gretag Macbeth SpectroEye), and their CIE 1964 10° xy chromaticities under equal energy white illuminant are shown in the inserted horseshoe diagrams. More details of the color system are documented elsewhere (MacAdam, 1974, 1978), but the system was shown to be effective to capture color categorization patterns, where discrete and perceptually uniform sampling of colors are key considerations (e.g., Boynton & Olson, 1987; Boynton, Maclaury, & Uchikawa, 1989).

### Observers

Six observers participated in Experiment 1-1 (A.H., K.S., K.U., M.M., Y.E., and Y.Y.). Three of the six observers (K.S., K.U., and Y.Y.) were further recruited for Experiment 1-2 and for Experiment 2. They were all screened to have normal color vision using Ishihara pseudoisochromatic plates. All observers were native Japanese speakers. Raw categorical responses were thus obtained in Japanese, which were translated to English for reporting purposes. The translation was straightforward as English speakers and Japanese speakers have largely equivalent basic color categories (Uchikawa & Boynton, 1987).

## Experiment 1-1

### Illuminant condition

**Figure 2.**
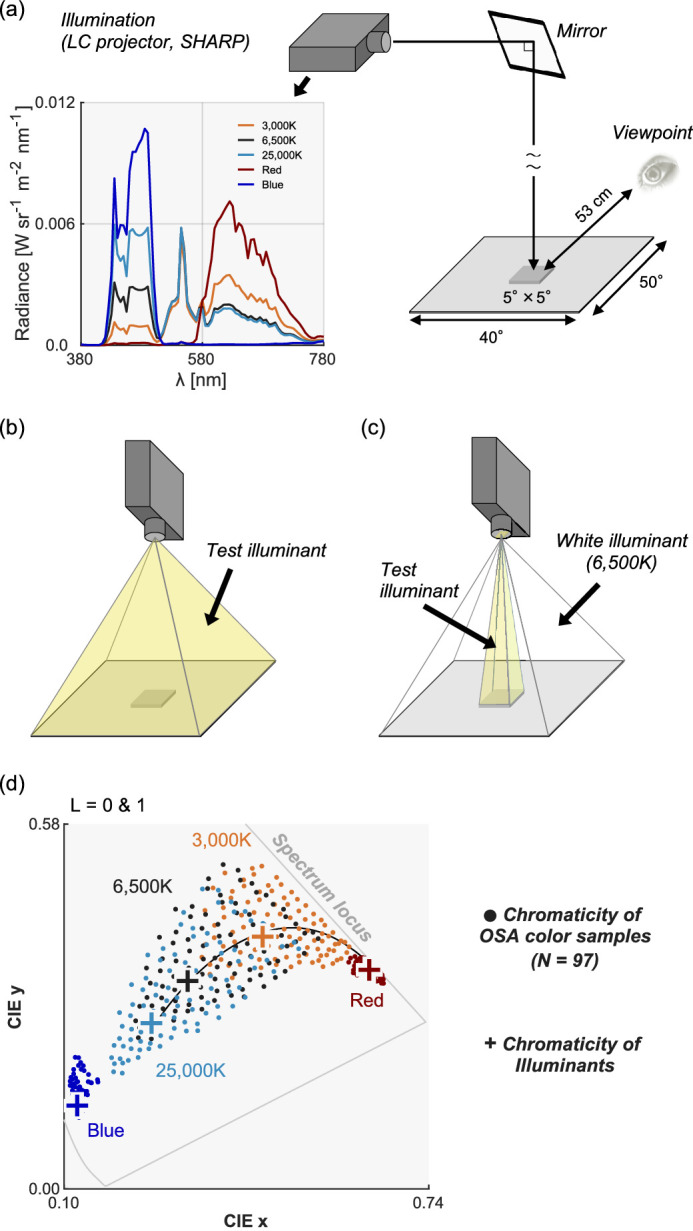
(a) Experimental setup in Experiment 1-1. The 5° × 5° test color sample was presented on a uniform gray surrounding. For the whole illuminant condition, both the test color sample and the surrounding gray region were illuminated while for the spot illuminant condition only the test sample was lit by the light source. (b) Whole illuminant condition. Test color sample and surrounding region are both illuminated by the test illuminant. (c) Spot illuminant condition. Only the test color sample is illuminated by the test illuminant and the surrounding region is illuminated by a 6,500K white light. (d) The influence of illuminant change on the CIE 1964 xy chromaticity of 97 color samples (*L* = 0 and 1). The plus symbols depict the chromaticities of test illuminants that varied along and beyond the black body locus shown by the black solid curve.

For test illuminations, we used five illuminant spectra generated by a liquid crystal projector (SHARP LCP). Figure 2a shows their spectral compositions together with the experimental setup. The light emitted from the projector reached the test surface via the reflection of a mirror, and the light source was not directly visible to observers. Observers sat 53 cm from the test sample. For each trial, the experimenter carefully mounted a test color sample on a 5° × 5° square pedestal with a few mm height from a 50° × 40° gray surrounding surface (lightness level equivalent to OSA *L* = −2). There were two ways to illuminate the test color sample. For (i), the whole illuminant condition as shown in Figure 2b, a color sample and the surrounding gray region were both illuminated by the test illuminant. This standard condition allowed us to measure the categorical patterns when categorical color constancy works to some extent. However, to quantify the degree of color constancy, we thought that it was desirable to know the categorical patterns without color constancy as a baseline measurement. This is a problem especially for color samples that are located around the center of a large color category, because their category may not change even under large illuminant changes. In this sense, some colors have some baseline stability even without any color constancy mechanism. Thus, in addition to the standard whole-illuminant condition, we used (ii) the spot illuminant condition where only the test color sample was illuminated by the test illuminant while the surrounding region was illuminated by a 6,500K white light as depicted in Figure 2c, which was made possible by virtue of the use of an LC projector. This spot-illuminant condition fully eliminated cues to the color of test illuminant and created a condition where the color of the test illuminant appears to belong to the color of the test sample, thereby allowing us to measure color naming patterns under the test illuminant without color constancy. The naming patterns in the spot illuminant condition effectively defined the baseline for each color sample. The square pedestal was used to raise the color sample a few mm which minimized the amount of illuminant leaking into the surrounding region. We found this trick useful to make the test color sample appear as if the spectral reflectance of the surface changed when the test illuminant changed (i.e., no constancy).

To show the magnitude of each illuminant change, Figure 2d shows the chromaticities of 5 test illuminants and the associated chromatic distribution of the 97 color samples at a mid lightness level (*L* = 0 and 1) under each illuminant. The color temperatures 3,000K and 25,000K were selected as they were around the edges of the natural daylight variation (Morimoto, Zhang, Fukuda, & Uchikawa, 2022). The red and blue illuminants were achieved by activating only red or blue phosphors of the projector, respectively. Thus, they had particularly high stimulus purity, under which color samples had chromaticities tightly clustered near the spectrum locus. These illuminants were chosen to investigate the limit of categorical color constancy outside the natural illuminant variation.

### Procedure

Observers named 424 color samples under 10 different illuminant conditions (5 illuminant colors × whole and spot conditions), using 1 of 11 basic color categories: red, green, yellow, blue, brown, orange, purple, pink, gray, white, and black. The basic color terms were used for the following reasons. First, the purpose of this study was to investigate invariant categorical regions across illuminant changes; thus, to address this question we needed to have a high enough number of responses for each color category. If instead we used a greater number of categories, we presumably could not obtain enough responses to analyze behaviors of rare categories across illuminant conditions. Second, we focused on color terms that people naturally use for communications in their daily lives.

One session consisted of 4,240 responses, and all observers completed 2 sessions. After the categorization of all 424 color samples under each illuminant condition, observers were additionally asked to select a focal color for each color category. We note that the definition of focal color varies across studies as described in detail elsewhere (e.g., Witzel, 2019), but this study uses the term as the best example of a color category. There was no time limit in each trial. Observers first adapted to a test illumination for 5 minutes via the reflection from the gray surrounding region with no test color sample presented. Color samples were presented one at a time in a random order. The order of illuminant condition was also randomized.

### Results and discussion

#### Color naming patterns in the OSA color system

**Figure 3.**
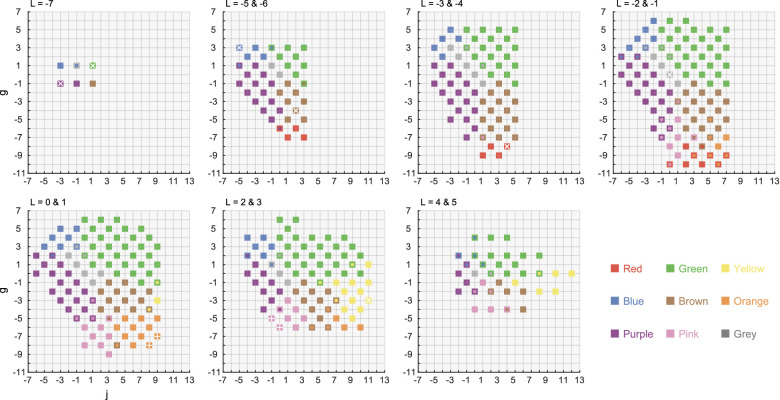
Color naming results under 6,500K whole-illuminant for one observer AH. Data points marked by two colors show the color name used for the first session (outer color) and for the second session (inner color). The color sample chosen as the focal color is depicted by a white cross (if consistent across two sessions) or a white plus (if inconsistent).

Figure 3 shows the color naming results under the 6,500K whole-illuminant condition for a representative observer AH. Each data point was colored by the color term used by the observer. A bicolored data point show the color name used for session 1 (outer color) and for session 2 (inner color). Color samples selected as focal colors are additionally marked by a white cross (if consistent across sessions) or a white plus (if not). The color categories spread across the OSA color system, and it is shown that some color categories (e.g., green) were used more than others (e.g., red, gray). The black and white categories were not used by this observer. Overall response patterns were consistent across tested observers, and noteworthy variations were not observed.

**Figure 4.**
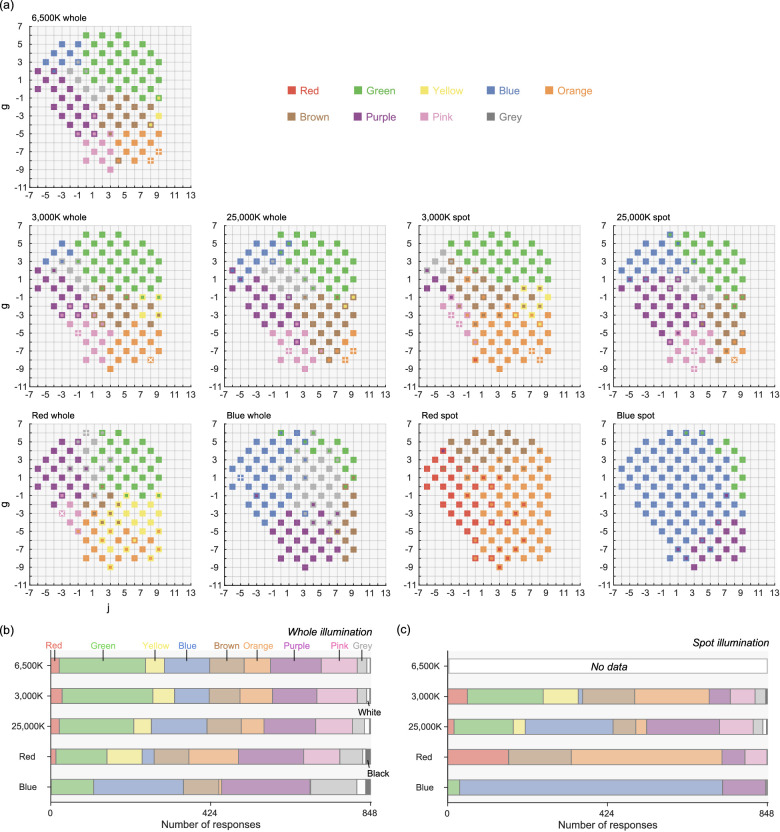
(a) Color naming results at *L* = 0 and 1 under different illuminant conditions for observer A.H. The upper subpanel replots the result for the standard illuminant condition for comparison purposes. The left and right four subpanels depict results for the whole illuminant and spot illuminant conditions, respectively. Color samples that were selected as focal colors are marked by a white cross (if consistent across sessions) or a white plus (if inconsistent). (b, c) The number of categorical responses for the whole illuminant condition and spot illumination condition, respectively. The numbers were averaged across six observers.

Figure 4a shows how color naming patterns change in response to illuminant change. For brevity, we show only the results for 97 color samples at *L* = 0 and 1. The upper subpanel replots the standard illuminant condition (6,500K-whole) for comparison. A perfectly color-constant observer would show the same categorical response pattern regardless of illuminant conditions.

For the 3,000K-whole and 25,000K-whole conditions, color categories are largely similar to those in the standard illuminant condition. In contrast, for the red whole-illuminant, slightly more yellow and orange responses are seen around the lower part, and blue responses around the upper left region were replaced by purple responses, showing a bias toward the color of the test illuminants. In addition the blue whole-illuminant showed more prevalent blue and purple responses, showing a failure of categorical color constancy.

For the 3,000K-spot and 25,000K-spot conditions, although we still see a somewhat similar pattern to that of the standard illuminant condition, response patterns are more severely affected by the illuminant color than the whole illuminant conditions, as we had expected. Some color categories did not change, likely because the shift of chromaticity owing to illuminant change was not large enough to alter categorical responses for those color samples, showing their baseline tolerances. For red-spot and blue-spot illuminants, however, response patterns are largely different, where mostly red and orange or blue and purple responses were recorded, respectively. Comparing these patterns to those in red-whole and blue-whole conditions allows us to appreciate how well categorical color constancy holds in their respective whole illuminant conditions.

Figure 4b and 4c summarize the number of color terms used for the whole illuminant and the spot illuminant conditions, respectively, visualizing how the relative frequency of categories varies across illuminants. There were 848 responses in total for each illuminant condition (424 color naming × 2 sessions), and the numbers were averaged across all six observers. The number of responses naturally varies across color categories. For example, red and yellow categories were used much less frequently than colors such as green, purple and pink. Also, Figure 4c clearly visualizes that categorical patterns differ much from the whole illuminant condition, especially for the red and blue illuminants. The proportion of white and black responses were only 1.470% and 0.723% on average across observers, and thus we excluded these categories from subsequent analyses. We suspect that the small percentages of white and black responses are due to the limited number of achromatic colors in the OSA system.

#### Categorical centroids

**Figure 5.**
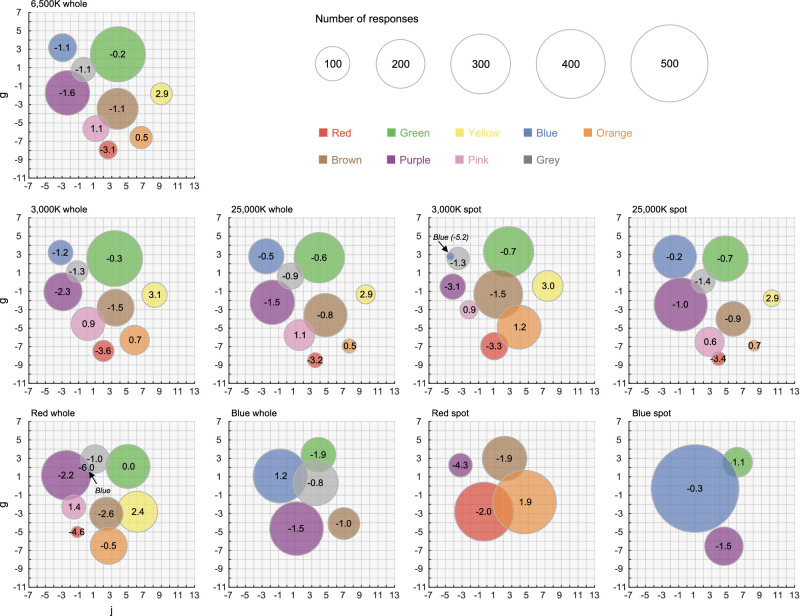
Centroids of color categories for observer AH. The size of each circle depicts the number of responses in the category. The value in each circle is the *L* value of each centroid.

We next quantified the magnitude of categorical shifts induced by the illuminant change. To define a representative location for each color category, we computed their centroids in the following way that is in accordance with past studies (e.g., Boynton et al., 1989). For each observer, there were in total 848 responses, each labeled by a color naming. Then, for a specific color category, for instance red, we took all color samples named red, and calculated the mean *j*, *g*, and *L* values across the samples. The calculation was done separately for each illuminant condition. Figure 5 shows the centroids of color categories for each illuminant condition and the value in each circle shows the *L* value of the centroid. The size of each circle corresponds to the number of responses that went into the computation of the centroid. Then, for each color category, we calculated the OSA color difference ΔUCS between standard illuminant (i.e., 6,500K-whole) and each illuminant condition using Equation (1), where suffixes 1 and 2 indicate the standard illuminant and each illuminant, respectively. The weighting of square root two is applied to *L* values to account for the geometrical asymmetry in the color system (MacAdam, 1974).

(1) $$\begin{array}{ccc} & & \;\Delta UCS\;\\ & & \;\;=\sqrt{{\left(\sqrt{2}L_{1}-\sqrt{2}L_{2}\right)}^{2}+{\left(j_{1}-j_{2}\right)}^{2}+{\left(g_{1}-g_{2}\right)}^{2}}\; \end{array}$$

**Figure 6.**
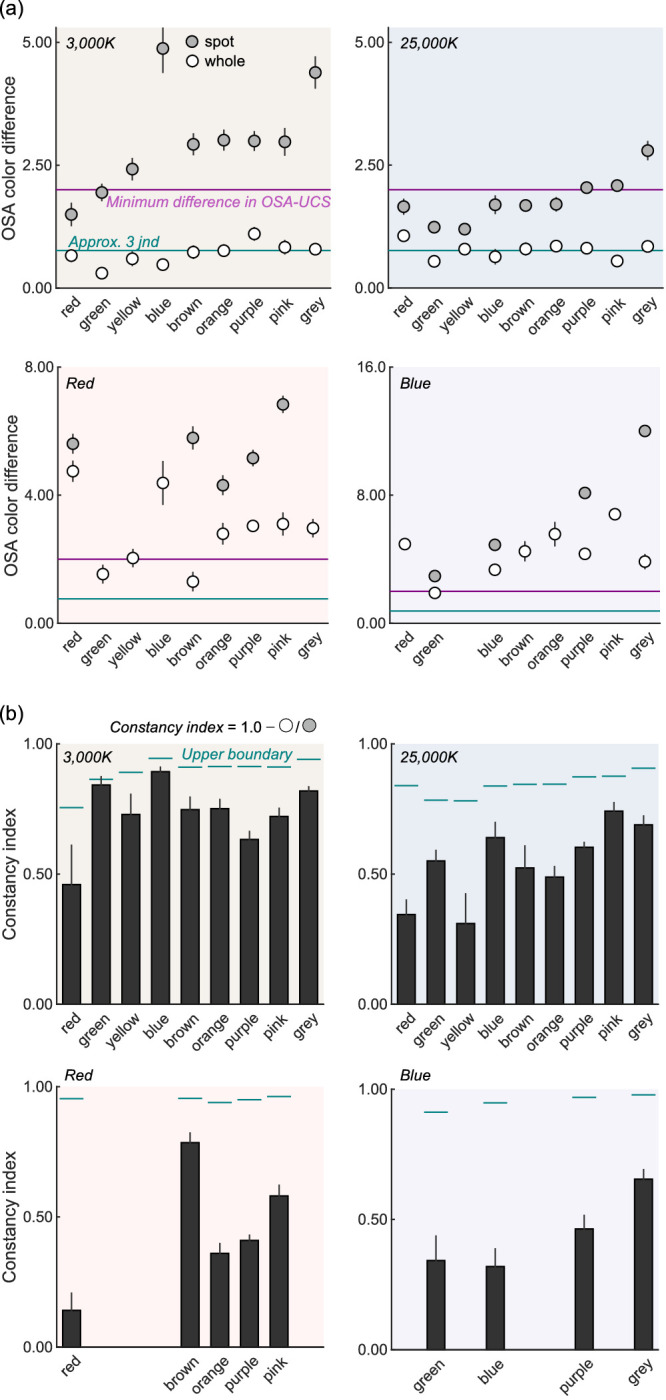
(a) The shift of categorical centroid in the OSA unit between the 6,500K whole-illuminant condition and other illuminant conditions. The magenta horizontal line shows the minimum difference that can be defined in the OSA-UCS. The cyan horizontal lines indicate roughly 3 jnds, estimated based on relation between ΔUCS and DE2000 (see main text for details). (b) Constancy indices defined by the ratio of ΔUCS between the whole illuminant and the spot illuminant condition subtracted from one. The value one means perfect color constancy and zero shows no color constancy. The error bars show ± 1.0 standard error across observers in both panels.

Figure 6a shows the ΔUCS values for whole and spot illuminant conditions, averaged across six observers. The absence of data points means that no observer used the color name for the condition. For data points where one or more observers showed no response, we calculated the average without those observers. In the OSA-UCS, neighboring colors have at least 2.0 ΔUCS (shown by horizontal magenta lines). We see that the ΔUCS values in the whole illuminant condition are very small for the 3,000K and 25,000K conditions, where values were lower than 1.0 in most categories and, thus, comfortably smaller than the minimum difference in the color system. As expected, the ΔUCS values in spot illuminant condition were always larger than the whole illuminant condition.

Furthermore, to have a more concrete idea as to the magnitude of these shifts, we analyzed how ΔUCS values relate to another commonly used color difference metric DE2000 defined in L*a*b* color space (Luo & Rigg, 2001). For this, we calculated ΔUCS values and DE2000 values for all possible pairs of 424 OSA color samples (89,676 pairs). It was found that ΔUCS and DE2000 are linearly correlated, *r* = 0.906, *p* < 0.00001, and that 0.255 ΔUCS approximately corresponds with 1.0 DE2000, which is often interpreted as one just noticeable difference (jnd). As shown by the cyan horizontal line in Figure 6a, 3 jnds were found to be roughly equal to the magnitude of shifts in the 3,000K and 25,000K whole illuminant conditions. However, it should be stressed that this relation between two color metrics is only a loose approximation and should not be taken as a generic conversion for other uses.

For the red and blue whole illuminant conditions, the color shifts are much larger than the 3,000K and 25,000K conditions, except for the yellow category in the blue whole illuminant condition that was not used by any observer. And for spot illuminant conditions, some categories were used by no observers, shown by the absence of data points.

To summarize these in a more quantitative way, we next calculated constancy indices using Equation (2). Here *a* and *b* show ΔUCS values in the whole illuminant condition and ΔUCS values in the spot illuminant condition, respectively. The value one and zero of the index means perfect and no constancy, respectively.

(2) $$\begin{array}{c} CI=1-a/b\; \end{array}$$

Figure 6b shows the indices for all illuminant conditions. Some categories were left blank because it was not possible to compute the index for these categories because of the absence of data points (as seen in Figure 6a). It was shown that categorical constancy holds well for 3,000K and 25,000K, although there are some areas of improvement, especially in the 25,000K condition. For the red and blue conditions, color constancy was found to hold for some categories to some extent. It should also be noted that the blank data do not mean no color constancy. If anything, they show high color constancy in a sense that no responses were recorded in the spot-illuminant condition, but they emerged in the whole-illuminant condition thanks to color constancy. We also considered that the constancy index is likely limited by our absolute color sensitivity (jnd). Based on this thought we computed the upper-bound of constancy indices by taking the ratio between 0.255 ΔUCS (1.0 jnd as estimated elsewhere in this article) and ΔUCS in the spot illuminant condition*,* which was then subtracted from one. This is shown by the cyan lines in Figure 6b. A constancy index between this line and 1.0 should be collectively interpreted as perfect constancy.

#### Focal colors

Next, we analyzed the focal colors, which might serve functional significance in mechanisms of categorical color perception because they are likely the first colors that come to our mind when we communicate using color terms. For the communication to be effective, however, the focal color needs to be shared across individuals at least to some extent and also needs to be robust across lighting environments. Based on these ideas, we have analyzed the locations of the focal colors to see i) how they vary across individuals and ii) how they change depending on the illuminant condition.

**Figure 7.**
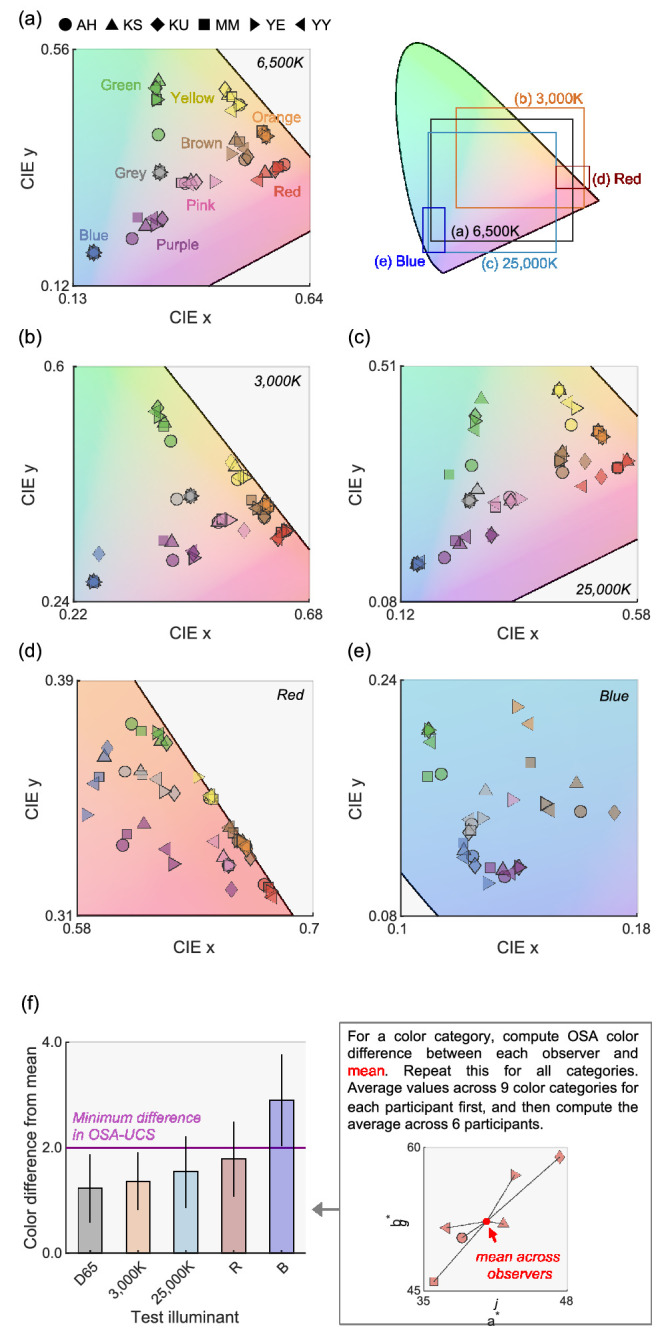
CIE 1964 xy chromaticity of color samples chosen as a focal color for (a) 6,500K, (b) 3,000K, (c) 25,000K, (d) red, and (e) blue illuminants (whole-illuminant condition). The color of the symbol depicts the color category and the shape shows observers. Each panel shows different *x* and *y* ranges, which are collectively shown in the inserted diagram next to (a). (f) Color difference in OSA unit between mean across observers and each observer. The value was averaged across color categories and then across observers. The magenta line shows ΔUCS = 2.0, corresponding with the minimum difference in the OSA-UCS*.* The error bars show ± 1.0 standard error across color categories.

Here, to see directly what chromaticities were chosen as focal colors under each test illuminant (whole-illuminant) we plotted them in the CIE 1964 xy chromaticity diagram as shown in Figures 7a to e. The shape of symbols depicts observers. For each panel, the plotted *xy* ranges differ as shown in the diagram next to Figure 7a. Roughly speaking, we see trends that focal colors are clustered somewhat closely for the 6,500K, 3,000K and 25,000K conditions. For the red and blue illuminant conditions, the variation across observers seems slightly higher. However, it is curious that many color categories emerge even for these extreme red and blue illuminants which push all chromaticities toward very small regions in a color space close to the spectrum locus. In contrast, for spot illuminant conditions where no color constancy holds, as shown in Figure 5, only limited categories were recorded for red and blue illuminants, meaning that the emergence of categories under the whole-illumination condition proves that this condition allows color constancy.

To quantify the variation of focal colors across observers, we calculated the mean ΔUCS between mean focal color point and focal color for each observer. The computation was done separately for each category first, and then took the average across categories. The right-hand part of Figure 7f also describes this computation. We see that the variation across observers is smaller than the minimum difference in the color system for natural illuminants (6,500K, 3,000K, and 25,000K) and red illuminant, suggesting that focal colors are relatively stable across observers for these illuminants. One-way repeated-measures analysis of variance (ANOVA) revealed that the main effect of illuminant conditions (6,500K, 3,000K, 25,000K, red, and blue) is significant, *F*(4, 20) = 14.4, *p* = 1.1 × 10^−5^. Multiple comparisons with Bonferroni's correction reported the following relation: 6,500K, 3,000K, 25,000K, and red < blue. In other words, other than the blue illuminant, the stability of focal color across observers was roughly equal.

These analyses generally supported the idea that focal colors were stable across at least the six tested observers. Next, we tested whether focal colors were stable across illuminants. For this, we first calculated the consistency of color category across illuminants for each color sample. For each color sample, we had 10 responses (5 illuminant colors × 2 sessions). Then, the consistency was defined as the proportion that the same color category was used for the color sample. For example, if the color sample was named by the same category 7 times out of 10 responses, this color sample had 70% consistency.

**Figure 8.**
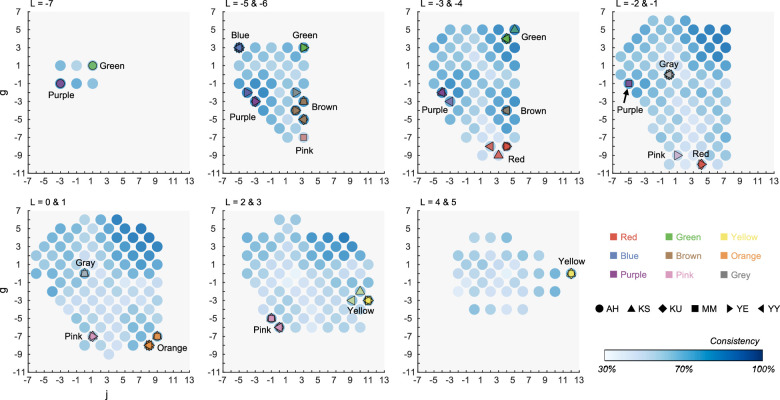
Consistency across five illuminants for focal colors and nonfocal colors in the whole illuminant condition. The values were averaged across six observers. For each color sample, there were 10 responses (5 illuminant colors × 2 sessions). The consistency was defined by the proportion that a color sample was called by the same category. Colored symbols show focal colors selected in the 6,500K whole-illuminant condition.

In Figure 8, this consistency is shown as the heatmap where darker blue indicates higher consistency.

The colored symbols show the location of focal colors under 6,500K, and the shape of symbols depicts observers. Roughly speaking, it is shown that focal colors are almost absent around the color samples that have particularly low consistency. Instead, they tend to fall on to color samples that have relatively high consistency though the exact trend depends on color categories. We see that this observation holds particularly well for categories such as green, purple, or brown.

We next computed the average consistency separately for focal color samples and nonfocal color samples. We first fixed a color category, and then we collected color samples that were selected as the focal color at least once under 6,500K and calculated mean consistency across the color samples. For nonfocal colors, we needed to define which color sample belonged to which color category, as the color naming varies across illuminant conditions. Each color sample was classified to one color category based on the mode across 10 responses (i.e., color name that was used most frequently for the color sample).

**Figure 9.**
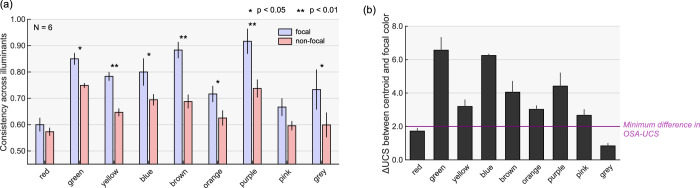
(a) Consistency across illuminants for focal color samples that were selected at least once as a focal color in 6,500K condition and nonfocal color samples that were never selected as a focal color. The symbols * and ** indicates pairs where significant differences were found (Bonferroni's correction applied). The error bars show ± 1.0 standard error across observers. (b) OSA color difference between centroid and focal color for each color category. The magenta line shows the minimum difference in the OSA color system. The error bars show ± 1.0 standard error across observers.

Figure 9a shows the consistency for focal color samples and nonfocal color samples. A two-way repeated-measures ANOVA confirmed the significant main effect of the focality, *F*(1, 5) = 73.6, *p* = 0.000355, focal > nonfocal, and the color category, *F*(8, 40) = 6.99, *p* < .00001. The interaction between the focality and the color category was significant, *F*(8, 40) = 2.85, *p* = 0.0132. Multiple comparisons by Bonferroni's correction showed that the focal color had higher consistency than nonfocal colors in most color categories as shown by asterisks in Figure 9a. We next compared the consistency between color categories and found significant differences for the following categorical pairs: for focal colors (red < green, brown, purple; pink < brown, purple) and nonfocal colors (red < green, purple; green > pink, grey; purple > pink, grey). To summarize, these analyses suggested that focal colors are more stable across illuminants than nonfocal colors, for most color categories.

One remaining question is why focal colors are stable. One possibility would be that focal colors are simply located near the centroid of the color category, and thus illuminant changes are less likely to push them outside the category. To test this idea, we calculated ΔUCS between the centroid and focal color, as shown in Figure 9b. The calculation was first done for each observer independently and averaged across two sessions. Then the ΔUCS values were averaged across observers. Rather surprisingly, we found that in most cases ΔUCS values are substantially higher than the minimum difference, indicating that focal colors do not necessarily coincide with categorical centroids. We see smaller ΔUCS values for red and gray categories, but the number of responses were lower in these categories. For these small color categories observers did not have an option to pick a color sample far from the categorical centroid as the focal color, which necessarily led to a small ΔUCS value here. Thus, these observations collectively reject the hypothesis that focal colors are stable merely because they are located around the center of color categories.

**Figure 10.**
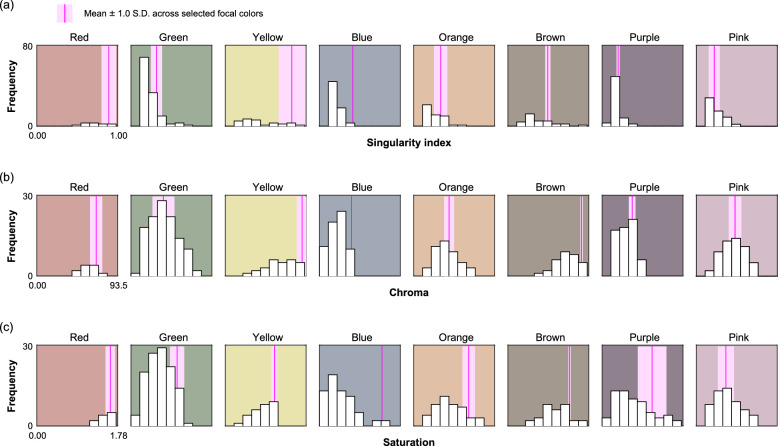
(a) Histogram of singularity index for all color chips that belong to each chromatic color category. Magenta line and shaded pink area shows mean ± 1.0 standard deviation across color chips that were selected as focal color at least once. (b) Histogram for chroma (C*_ab_ defined in L*a*b* color space). (c) Histogram for saturation (C*_ab_/L* defined in L*a*b* color space).

We next sought whether invariance of color categories and focal colors could be explained by the physical properties of color chips. Philipona and O'Regan (2006) suggested that color categories are more invariant for surfaces whose associated cone signals are stable across illuminant changes (the concept termed “sensory singularity”). Similarly, chroma and saturation were suggested to affect i) the consistency of color naming because physical reflected light changes less for highly saturated colors and ii) the choice of focal colors as saturated colors have high perceptual saliency (Reeves & Amano, 2020; Witzel, 2018). To test these metrics, we first computed the singularity index (using a MATLAB source code provided by Witzel, Cinotti, & O'Regan, 2015), chroma (C*_ab_ defined as the Euclidean distance from the white point in La*b* color space) and saturation (C*_ab_ / L*) for all 424 color chips. Then, we computed the correlation coefficient between each metric and the consistency map in Figure 8. We found no significant correlation with the singularity index, r = −0.0097, *p* = 0.842, close to the value reported in Olkkonen et al. (2009), and chroma, r = 0.0856, *p* = 0.0783. However, there was a weak but significant correlation with saturation, r = 0.231, *p* =1.54 × 10^−6^. Thus, these invariant color regions could be at least partially explained by the saturation of the color chips. Furthermore, we analyzed whether observers tended to choose a color chip that has a particularly high metric value as a focal color. Using responses in 6,500K whole-illuminant condition, we classified each of 424 color chips into a single color category based on the mode color category across 12 responses (2 repetitions × 6 observers). Figure 10 shows a histogram of each metric for chromatic categories. The magenta line and pink shaded area show mean ± 1.0 standard deviation computed over color chips that were selected as a focal color at least once in 6,500K whole-illuminant condition. Looking across all subpanels, we notice that focal colors in some categories such as red, yellow, or blue align closely with the right edge of the histogram, suggesting that the observers’ selection of focal colors was potentially guided by these metrics. However, for other categories such as green, orange, purple, and pink, this observation does not hold. To sum, these metrics based on low-level photoreceptor signals (singularity index) or perceptual metrics (chroma and saturation) do not completely explain the stability of focal colors and additional factors are likely to play a role, but they are candidate reasons for why focal colors are stable.

## Experiment 1-2

### Condition and procedure

**Figure 11.**
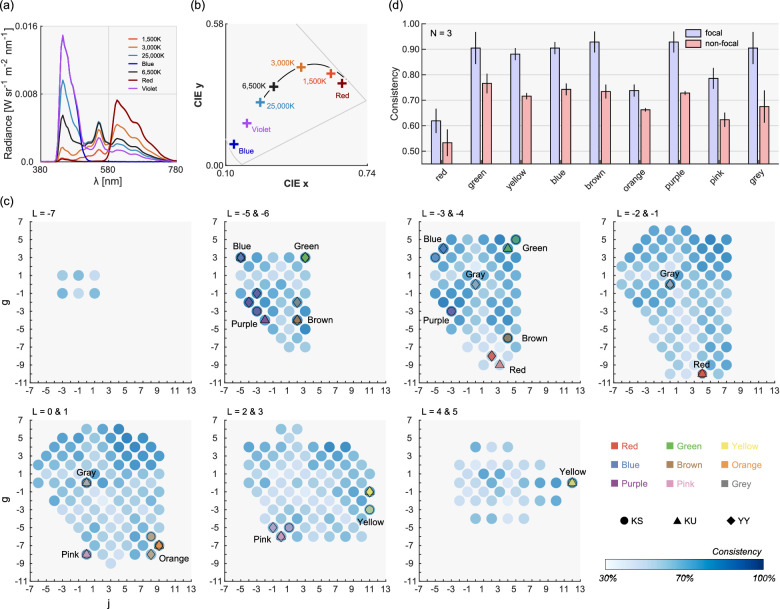
Experimental setup and results in Experiment 1-2 where a large number of illuminants were used compared with Experiment 1-1. (a) Seven Illuminant spectra and (b) their chromaticity in CIE1964 xy chromaticity diagram. (c) The map of categorical consistency across illuminants for all color samples. Colored symbols indicate focal colors and shapes indicate different observers. (d) Consistency of color naming across illuminants for focal colors and nonfocal colors. The error bars show ± 1.0 standard error across three observers.

One major finding in Experiment 1-1 was that invariant categorical regions coincide with focal color locations. However, the consistency values shown in Figure 9a directly depend on the number and variation of illuminants we used. We thus felt it would be desirable to validate this main finding using an expanded set of illuminants and decided to run a supplemental experiment. Figures 11a and b show the spectra of seven illuminants used in this experiment. Two additional light sources were 1,500K and violet, whose chromaticities were located between 3,000K and red and between 25,000K and blue, respectively. A different three-primary liquid crystal projector was used in this experiment (EPSON, EMP-54, spatial resolution 800 × 1300, horizontal scanning frequency 15–92 KHz, vertical scanning frequency 50–85 Hz). We used only the whole illuminant condition. Other experimental conditions and procedures were identical to those in Experiment 1-1.

### Results and discussion

Figure 11c shows the consistency map across illuminant conditions for all color samples. For each color sample, there were 14 categorical responses (7 illuminants × 2 sessions) from which we calculate the consistency of color categories. The patterns are similar to Experiment 1-1 as shown in Figure 8, but the trend that focal colors coincide with highly consistent regions seems clearer. There was no significant correlation between this consistency map with the singularity index, r = 0.0435, *p* = 0.371, but we found low but significant correlations for chroma, r = 0.110, *p* = 0.0240, and saturation, r = 0.200, *p* = 3.32 × 10^−6^. Figure 11d shows the averaged consistency for focal colors and nonfocal colors, which again shows very similar trends to Experiment 1-1 (Figure 9a). A two-way repeated-measures ANOVA confirmed that there is a significant main effect of focality, *F*(1, 2) = 100.4, *p* = 0.00981, and color category, *F*(8, 16) = 9.40, *p* = 0.000089. The interaction between the two factors was not significant, *F*(8, 16) = 1.01, *p* = 0.466, meaning that focal color had higher consistency than nonfocal color for all color categories. To summarize, this additional experiment consolidated the main finding in Experiment 1-1.

## Experiment 2: Asymmetric color matching

One major finding in Experiment 1 was that categorical color constancy holds remarkably well, especially under 3,000K and 25,000K. However, there are at least two potential reasons for this. First, it is possible that the color appearance of color samples did not change much under test illuminants compared with the 6,500K condition, which consequently induces little categorical change. This first possibility shows that it is the appearance-based color constancy that held well, and the invariance of color category is simply a byproduct. A second possibility, which we think is more interesting, is that although the color appearance shifted influenced by the illuminant change, color categories did not change because they are inherently more tolerant than color appearance. This second possibility highlights the advantage of using a color category in color constancy mechanisms. Thus, to contrast the difference between appearance-based color constancy and categorical color constancy, Experiment 2 directly measured how much color appearance changed under a test illuminant using an asymmetric color matching task well-established in the field (e.g., Brainard & Wandell, 1992).

### Conditions and procedure

**Figure 12.**
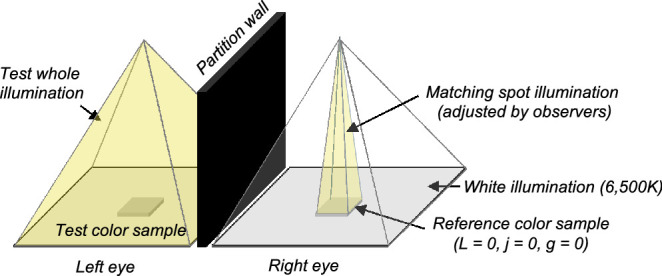
Experimental setup for asymmetric color matching. The left side is a test scene where the test color sample was illuminated by one of 5 illuminants used in Experiment 1 (6,500K, 3,000K, 25,000K, red, and blue). For the test color sample, we used a color sample named gray under each test color illuminant. The right hand had a reference color sample (*L* = *j* = *g* = 0) that was illuminated by the spot illuminant, whose R, G, and B values were independently adjusted by observers until the appearance of test and reference color samples matched.

Figure 12 shows the scene configuration in Experiment 2. The scene consists of two parts. On the left side, a test color sample was lit by a whole illumination. On the right side, a reference color sample (*L*, *j*, *g* = 0) was lit by a spot illumination. For a surrounding region on both sides, we used the same gray color paper as Experiment 1. Observers viewed the scene with their forehead attached tightly to the black partition wall so that the right eye sees only the matching scene and that the left eye sees only the test scene (dichoptic presentation). For the test whole illumination, we used the five illuminants used in Experiment 1-1, namely 6,500K, 3,000K, 25,000K, red, and blue illuminants. For the test color sample, we used color samples named gray under each test illuminant, which allowed us to measure how the appearance of categorical achromatic points shifted depending on the illuminant color. There were several reasons to use the gray category as a test color. First, to measure how the appearance of color chips in the specific color category differ across different test illuminants (3000K, 25,000K, red, and blue), the color category must be used i) under all illuminants to allow comparison across the illuminants and ii) by all observers so that we can use the consistent color category for all observers. There was also a practical reason that some color categories have too many color chips (e.g., green, purple, and blue) as matching experiments takes a long time. These criteria together suggested that gray would be a good category to test. The gray color samples were located on *L* = 0 and 1 planes for KU (51 samples in total across 5 test illuminants) and *L* = −1, 0, and 1 planes for Y.Y. (61 samples) and K.S. (31 samples). The observer's task was to change the color of matching spot illumination by adjusting the intensity of R, G, and B phosphors of the projector independently until the appearance of the reference and test color sample matched. Two matchings per color sample were obtained. There was no time limitation for each trial.

### Results

**Figure 13.**
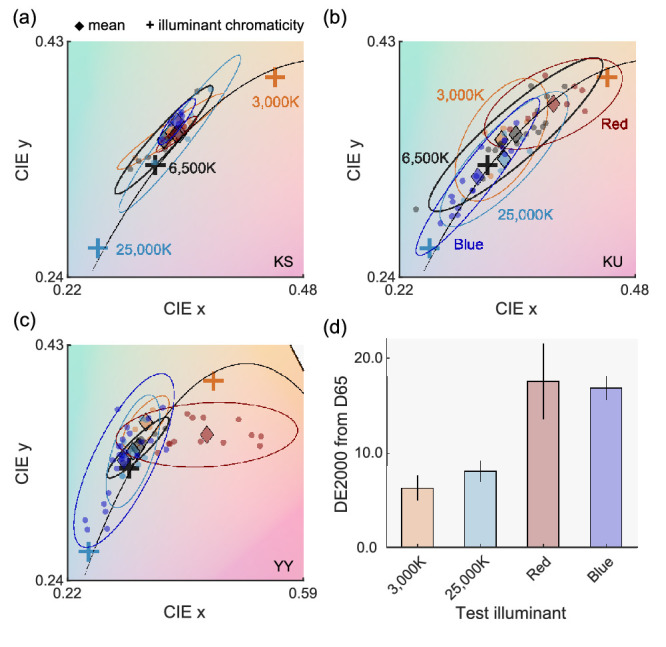
Results of asymmetric matching for (a) observer K.S., (b) K.U., and (c) Y.Y. Each small circle shows one matching. The diamond symbols depict the average across all matchings. Ellipses were fitted to data points with 95% confidence intervals. Notice that the *x* axis range is wider for (c) than (a) and (b). (d) DE2000 between mean matching points for 6,500K condition (gray diamond in a–c) and other illuminant conditions (colored diamonds). Each bar shows an average across three observers and error bars show ± 1.0 standard error across observers.

Figures 13a to c shows matching results for each observer. Each colored small circle depicts the averaged matching point across two repetitions and diamonds show mean chromaticity across all settings for each illuminant. The ellipse was fitted to cover the data points in each illuminant condition with 95% confidence intervals. For Figure 13a (observer K.S.), the matching points were clustered closely across all illuminant conditions. In contrast, for Figure 13b (K.U.) and Figure 13c (Y.Y.), matching points in each illuminant condition were separated from each other. We quantified this separation using DE2000 between 6,500K (grey diamond) and other illuminants (colored diamonds) as shown in Figure 13d. Each bar shows an average across three observers. A one-way repeated-measures ANOVA showed that the main effect of illuminant color is significant, *F*(3,6) = 6.48, *p* = 0.0260, but multiple comparisons confirmed that there is no significant difference for any pair (Bonferroni's correction). The mean DE2000 value across participants was at least 6.21 (3,000K condition) and approximately 17 for the red and blue illuminant conditions. These results show that shifts of color appearance are 17 times larger than one jnd, meaning that the appearance of the color samples named gray under red and blue illuminants are visibly different from the appearance of color samples named gray under 6,500K. Despite such large appearance differences, those color samples were still all named gray. To sum, these results suggest a category-based constancy is substantially more robust than appearance-based constancy. This greater robustness of categorical color constancy occurs potentially because categorical color naming is more tied to the judgment about the inherent property of an object as opposed to the color appearance of the object (so-called hue and saturation match vs. paper match; see e.g., Reeves, Amano, & Foster, 2008 and Radonjic & Brainard, 2016 for further discussion).

## General discussion

The present study measured categorical color naming patterns using a fairly large number of color samples placed under an extensive set of illuminants, which allowed us to comprehensively quantify the degree of categorical color constancy. A spot illuminant methodology was introduced and shown to be effective to measure categorical patterns under different illuminants while silencing color constancy mechanisms. We further conducted an asymmetric matching experiment to measure how much color appearance shifted within the color samples that were all named gray. We found that categorical color constancy works generally well, and invariant categorical regions curiously overlap with focal color regions. These results are largely in line with past studies (Olkkonenn et al., 2009; Olkkonen et al., 2010). Furthermore, we found that focal colors do not necessarily locate around the centroid of color category, suggesting that focal color is stable for a nontrivial reason. Metrics such as sensory singularity index, chroma, and saturation explained the stability of focal colors to some extent. It was also revealed that the appearance of a set of color samples that were all named gray change largely across color spaces (except observer K.S.) in response to illuminant changes, emphasizing that it is the nature of the categorical color system that keeps the high degree of constancy. We suggest this is at least partially because when reporting a color name, we naturally base our judgments on the intrinsic physical property of a surface rather than the color appearance (Radonjic & Brainard, 2016; Reeves et al., 2008). The knowledge as to which regions in a color space remain categorically invariant is valuable in basic research, but it could be also used to inform ergonomic industrial designs. For example, color designers can pick a color from the invariant regions in the color space so that the color category of products stays the same under different lighting conditions.

Although we generally observed quite a high degree of categorical color constancy, constancy indices shown in Figure 6 were still substantially lower than the upper bounds. One potential reason might be that there was not enough information about illuminant influence in the scene. Although the gray surrounding area would provide a direct cue to the color of illuminant, having some color variations in surrounding context also might have improved color constancy (Maloney & Wandell, 1986; Morimoto, Kusuyama, Fukuda, & Uchikawa, 2021). Another candidate would be the color of test illuminants we sampled. It is worth noting that in a previous study where temporal daylight variation within a day was measured (Morimoto et al., 2022), we found that most daylights fell within the range between 4,500K and 20,000K. Thus, it is possible that the natural illuminants we used (3,000K or 25,000K) may have induced too great of illuminant changes. In retrospect, the use of less extreme correlated color temperature might have increased the degree of color constancy. However, conversely it may be surprising that a relatively high degree of color constancy was found even under these large illuminant changes.

We here list some limitations in the present study for future studies. First, OSA color samples were used in all experiments. We chose the system mainly because of their perceptual uniformity, but categorical naming patterns are inherently constrained by the geometry of the color space and we indeed observed that the number of responses differed from one color category to another as shown in Figures 4b and c. This observation suggests that the use of finer scale might be desirable around a category where smaller numbers of responses were recorded (e.g., red or yellow). Consequently, we should not put too much emphasis when interpreting results for these categories. For example, the focal colors were found to be close to the categorical centroids for small categories (Figure 9b), but this is more likely to stem from the property of stimuli than the nature of focal colors. Past studies also have emphasized the importance of controlling low-level visual information when measuring categorical influences (see Witzel & Gegenfurtner, 2011; Witzel & Gegenfurtner, 2015). Ultimately, the stimulus dependency of our findings is not exactly clear until other surface sets are tested, but it should be noted that Boynton et al. (1989) reported that color naming patterns did not differ in a significant way when Munsell color chips were used instead. Another limitation regarding stimuli is that illuminants we used were sampled from the black-body locus and its extension. Although Olkkonenn et al. (2009) and Olkkonen et al. (2010) found little differences between blue–yellow illuminants and red–green illuminants, it is an interesting question whether our findings hold for illuminants sampled at different regions in a color space. Furthermore, the number of observers recruited in this study is relatively limited. This is because we used a large number of color samples and illuminant conditions, and we aimed to collect a large number of responses per observer (in total 8,480 responses). However, consequently the present study does not directly address the individual differences in color categories, which is currently a missing piece in the field of categorical color perception to our knowledge. Finally, the ethnicity of observers in this study is limited to Japanese, and it is an open question how much findings translate to other observers who have different linguistic origins.

Because we have found the significance of focal colors, their potential roles in our categorical color system have been the center of discussion in the field. Several unique features of focal colors have been discussed and in some cases questioned. Are focal colors consistent across languages (MacLaury, 1997; Regier, Kay, & Cook, 2005; Kuehni, 2007)? Does the notion of representativeness explain the variability and universality of focal colors across languages (Abbott, Griffiths, & Regier, 2016)? Do focal colors seem to be more salient (Regier et al., 2007; Witzel & Franklin, 2014)? Are there similarities between unique hues and focal colors (Kuehni, 2001; Miyahara, 2003)? Moreover, in agreement with our findings, some studies reported the particularly high categorical constancy for focal colors (Olkkonenn et al., 2009; Olkkonen et al., 2010), whereas others found no evidence for this effect (Witzel, van Alphen, Godau, & O'Regan, 2016). In communication using color terms, focal colors may be particularly critical because they are the colors that people are likely to imagine first in their mind, for example when they are requested to pass a red coat. Thus, for efficient communication it is beneficial to have higher stability at focal colors. Curiously, a recent study showed that the categorical centroids when computed by weighting each color sample by its communication needs coincide well with focal colors (Twomey, Roberts, Brainard, & Plotkin, 2021), suggesting the pivotal role of focal colors in communication. The final remaining open question is why focal color is stable. We confirmed that it is not simply because they are at the categorical center. We also found that metrics such as the singularity index, chroma, and saturation are generally good candidates to explain high stability of focal colors, but for some color categories this observation did not hold. Here, we speculate that people choose to label certain regions in a color space as focal, and the results here suggest that one factor influencing the deployment of that label is the greater invariance of this region under illumination changes which could facilitate robust communication with others using color terms in an unstable world. If we instead have focal colors highly variable across illuminant changes, we may not have been able to rely on our color naming system. However, we emphasize that this explanation alone cannot be a whole story. As past studies have argued, the nature of focal colors and formation of color categories are inherently multidimensional because perceptual, biological and cultural factors are likely to interact in complex ways. Thus, a complete model of categorical color perception must integrate these diverse perspectives. Nevertheless, we believe that the present study sheds a further light on the perceptual aspect of categorical color constancy.
